# ABA Inducible Rice Protein Phosphatase 2C Confers ABA Insensitivity and Abiotic Stress Tolerance in *Arabidopsis*

**DOI:** 10.1371/journal.pone.0125168

**Published:** 2015-04-17

**Authors:** Amarjeet Singh, Saroj K. Jha, Jayram Bagri, Girdhar K. Pandey

**Affiliations:** Department of Plant Molecular Biology, University of Delhi South Campus, Benito Juarez Road, Dhaula Kuan, New Delhi, India; Institute of Genetics and Developmental Biology, Chinese Academy of Sciences, CHINA

## Abstract

*Arabidopsis* PP2C belonging to group A have been extensively worked out and known to negatively regulate ABA signaling. However, rice (*Oryza sativa*) orthologs of *Arabidopsis* group A PP2C are scarcely characterized functionally. We have identified a group A PP2C from rice (OsPP108), which is highly inducible under ABA, salt and drought stresses and localized predominantly in the nucleus. Genetic analysis revealed that *Arabidopsis* plants overexpressing OsPP108 are highly insensitive to ABA and tolerant to high salt and mannitol stresses during seed germination, root growth and overall seedling growth. At adult stage, OsPP108 overexpression leads to high tolerance to salt, mannitol and drought stresses with far better physiological parameters such as water loss, fresh weight, chlorophyll content and photosynthetic potential (Fv/Fm) in transgenic *Arabidopsis* plants. Expression profile of various stress marker genes in OsPP108 overexpressing plants revealed interplay of ABA dependent and independent pathway for abiotic stress tolerance. Overall, this study has identified a potential rice group A PP2C, which regulates ABA signaling negatively and abiotic stress signaling positively. Transgenic rice plants overexpressing this gene might provide an answer to the problem of low crop yield and productivity during adverse environmental conditions.

## Introduction

Abiotic stresses such as drought, high salinity, cold and heat adversely affect the plant growth and productivity. To combat various environmental cues, as an adaptive mechanism, various signaling cascades get activated in the plant cell leading to altered cellular function and response. Reversible protein phosphorylation mediated by kinases and protein phosphatases is one such adaptive cellular response to maintain a critical balance in phospho-regulation during normal and adverse growth conditions. Protein phosphatases have been known to mediate abiotic stress triggered signaling pathways and members of major phosphatase class, PP2C, have been variably implicated in a number of studies [1–6]. The phytohormone ABA is a major player in regulation of responses during abiotic stresses, especially drought and salt stresses. It has been well established that abiotic stress signaling is regulated by ABA dependent and ABA independent gene expression [7–10]. Group A of PP2C in *Arabidopsis*, which comprised of ABI1, ABI2, HAB1, HAB2, AHG1 and AtPP2CA, has been studied extensively and recognised as negative regulators of ABA signaling and responses [11–15]. Recent discovery of novel ABA receptors PYR/PYL/RCAR gene family of START proteins has further accentuated this connection and placed PP2Cs at centre stage of ABA signaling pathway where these receptors interact with PP2Cs to regulate abiotic stress responses and plant development [16–18]. In rice also, differential expression of several group A PP2Cs was reported under abiotic stresses and ABA treatment [6,19]. Recent studies have suggested that similar ABA signaling module is also working in crop plant rice and other plant species such as cucumber and tomato [20–22]. Rice ABA receptor OsPYL/RCAR5 was found to interact with group A PP2C, OsPP2C30 and a core ABA signaling unit was reconstituted, which also comprised of a ser/thr kinase SAPK2 [21]. Prior studies have shown high degree of sequence and structural conservation among protein phosphatases from different plant species [6,19]. The recent findings also indicate towards their functional conservation, especially for group A PP2Cs, from different plant species [23–24].

In this study, we are presenting one of the initial reports on detail functional characterization of a rice group A PP2Cs. We have identified a group A PP2C member from rice (*OsPP108/OsPP2C68*), which is highly up-regulated under ABA, salt and drought stress conditions. We generated constitutively expressing OsPP108 transgenic lines in *Arabidopsis* and seed germination based assays showed that transgenic plants are highly insensitive to high level of exogenous ABA. Expression level of ABA and stress related genes were altered in transgenic plants. Seed germination and stress tolerance assays for *Arabidopsis* adult plants showed that OsPP108 overexpression resulted in high degree of salt, drought and osmotic stress tolerance. Moreover, stomata movement assays under ABA treatment suggest that OsPP108 overexpression leads to ABA insensitivity even at adult stage. This study will help to comprehend the functional role of this PP2C gene in ABA signaling and abiotic stresses and can be utilized to develop transgenic rice plant with better stress tolerance and productivity.

## Materials and Methods

### Plant material, growth conditions and stress treatment

*Oryza sativa* ssp. *Indica* var. IR64 cultivar of rice was used for expression profiling. Rice seeds were sterilized and grown in culture room conditions according to Jain *et al*. (2004) [25]. *Arabidopsis thaliana* ecotype Columbia-0 was used for generation of transgenic plants. *Arabidopsis* seeds were treated with isopropanol for 5 minutes followed by 2% sodium hypochlorite for 10 min, washed five times with sterile water, and plated on MS medium solidified with 0.8% agar for aseptic growth and incubated at 4°C for 4 days under dark conditions. Seeds were grown under long-day conditions (16h-light/ 8h-dark cycle) in growth room. For growth in soil pots, seeds were sown on soilrite (1: 1: 1 ratio of vermiculite, perlite and Sphagnum moss) and regularly supplemented with Okada and Shimura (OS) nutrient medium [26]. For stress treatment, 7 days old IR64 rice seedlings were subjected to salt treatment by incubation in 250mM NaCl solution, dehydration stress by air drying on the Whatman paper and ABA treatment by incubation in 50μM (±) *cis*, *trans* ABA solution (prepared in absolute ethanol) at 28 ± 1°C along with respective untreated controls. Treated tissue was harvested at different time points and frozen immediately in liquid nitrogen. For stress treatment in *Arabidopsis*, 3 week-old seedlings grown on Murashige and Skoog (MS) basal medium were subjected to 300mM NaCl, 400mM mannitol and 50μM (±) *cis*, *trans* ABA treatment, at room temperature under white light and samples were harvested at different time points.

### Overexpression construct and transformation of *Arabidopsis*

To generate overexpression constructs, complete open reading frame (ORF) of *OsPP108* was PCR amplified using iProof high-fidelity DNA polymerase (Bio-Rad, Hercules, USA) and cloned at *Bam*HI and *Sal*I restriction sites of modified pCAMBIA1300 vector under the control of CaMV35S promoter. The binary construct was transferred to *Agrobacterium tumifaciens* strain GV3101 and used for *Arabidopsis* plant transformation by floral dip method [27]. T_0_ seeds harvested from these plants were screened on selection media (half strength MS media supplemented with 15μg/ml hygromycin) to obtain T_1_ plants. Transgenic plants were verified by segregation analysis in selection media (15μg/ml hygromycin) and then transferred to soil till maturity, to generate T_2_ and T_3_ generation, which were screened as homozygous transformants and used for further analysis.

### *In-silico* sequence and promoter analysis

Complete amino acid sequences of entire *Arabidopsis* group A PP2Cs were retrieved from The *Arabidopsis* Information Resource (TAIR) and for rice group A PP2Cs from RGAP-TIGR 7.0. Multiple sequence alignment was performed in ClustalX 2.0.8 with both *Arabidopsis* and rice sequences. Phylogenetic tree was constructed in MEGA5 with neighbour-joining, p-distance and pairwise deletion method. For promoter analysis, 1kb upstream sequence from translation start site of *OsPP108* was extracted from RGAP-TIGR and scanned in PlantCARE database for the presence of significant *cis*-regulatory elements.

### GFP-OsPP108 construct preparation, *Nicotiana benthamiana* leaf infiltration and confocal microscopy

Complete coding region lacking stop codon of *OsPP108* was amplified from stressed rice cDNA using iProof high-fidelity DNA polymerase (Bio-Rad). Amplified ORF was cloned in pENTR-D/TOPO vector (Invitrogen) by Gateway technology and subsequently mobilized to gateway compatible binary destination vector pSITE2CA [28] by LR reaction (Invitrogen). In pSITE2CA-GFP vector, 2XCaMV35S promoter controls the expression of cloned gene. Successful preparation of the construct was confirmed by PCR and sequencing methodologies. GFP-OsPP108 construct and empty pSITE2CA vector (control) were transferred to *Agrobacterium tumifaciens* GV3101::pMP90, which was then used to infiltrate 4–6 weeks old *Nicotiana benthamiana* leaves for transient expression. Subsequently, confocal microscopy for GFP and DAPI (4', 6-diamidino-2-phenylindole) localization was performed according to Mishra *et al*. (2013)[29].

### RNA isolation and cDNA preparation

Total RNA was isolated from rice and *Arabidopsis* tissues using Tri-reagent (Sigma, USA) according to manufacturer’s instructions. To nullify any genomic DNA contamination, isolated RNA was treated with RNase free DNAse I (NEB) and subsequently purified using NucleoSpin RNA clean-up kit (Macherey-Nagel, Germany). Quality and quantity of purified RNA was confirmed by reading absorption at 260nm, 230nm and 280nm at nano-spectrophotometer (Eppendorf). Ratio of 1.8–2.0 for A_260_:A_280_ and 2.0–2.3 for A_260_:A_230_ confirmed the high quality of RNA. Integrity of purified RNA was verified on MOPS buffer (Sigma, USA) RNA denaturing gel. cDNA was prepared from 1μg purified RNA in a 20μl reaction volume using high-capacity cDNA Archive kit (Applied Biosystems, USA) according to manufacturer’s instruction.

### Semi quantitative RT-PCR analysis

To confirm overexpression of *OsPP108* in *Arabidopsis*, semi-quantitative RT-PCR was performed for three *Arabidopsis* OX lines of OsPP108 along with WT (Col-0) with gene specific primer, using iTaq DNA polymerase (iNtRON Biotechnology, Korea) on BioRad C 1000 thermal cycler. The primer sequences used are as following OsPP108F: 5' CACCATGTCGATGGCGGAGGTGT 3', OsPP108 R: 5' CAAGGCGTTGCCTCGCCG 3'. For the quality of cDNA and as endogenous control, *ACTIN2* was amplified in parallel. 24 PCR cycles were used for amplification of both, *OsPP108* and *ACTIN2* genes.

### qPCR expression analysis

For qPCR analysis primers were designed preferably from 3’ end using PRIMER EXPRESS (PE applied Biosystems, USA) with default settings. Primers used in this analysis are listed in S1 Table. qPCR reaction was carried out in ABI Prism 7000 sequence detection system (Applied Biosystems, USA) using KAPA SYBR FAST Master Mix (KAPABIOSYSTEMS, USA). *ACTIN2* was used as endogenous control to normalize the cDNA variance among the samples. Relative expression was computed by ΔΔCt method according to Singh *et al*. (2012) [30].

### Germination and growth based phenotypic assays

About 100 seeds of three OsPP108^OX^ lines (L1, L2 and L4) along with WT were plated on half strength MS media plates supplemented with different concentration of ABA, NaCl and mannitol, and incubated at 4°C for 4 days for stratification and then transferred to growth room at 22°C, under long day (16hrs light/8hrs dark) condition. For vertical growth assays, plates were placed vertically on a rack. Percentage of seed germination (emergence of radicle) was scored until 5 days of growth. Cotyledon emergence was counted on the fourth day and fresh weight was measured after 7 days of growth. All the experiments were replicated thrice and average data from three observations with standard errors is presented.

### Salt, osmotic and drought tolerance analysis

*Arabidopsis* seeds of WT and OsPP108^OX^ were sown on pots containing soilrite and grown in LabTech (Daihann Inc, Korea) growth chamber with strictly regulated conditions (22°C, 60% humidity, 100 μmol m^-2^ s^-1^, 16h light/8h dark cycle). For salt stress and osmotic stress treatment, 3 week old plants were supplied with 300 mM NaCl and 400 mM mannitol solutions, respectively, after every 3 days, and plants were observed for 15 days for distinctive phenotype. Photographs were recorded after 10 days. Drought stress was subjected by withholding the water for 2 weeks and photographs were recorded after 12 days. For recovery, drought stress treated plants were resupplied with water and phenotype was recorded after 1 week. For the quantitative assessment of phenotype, various parameters such as survival percentage, total chlorophyll content and as a measure of efficiency of photosynthetic system, Fv/Fm ratio were recorded as described earlier [31]. To extract the chlorophyll, equal fresh weight of leaves were incubated in 80% acetone overnight in dark. Next day debris was pelleted down by centrifugation and absorption was recorded for the supernatant at 663nm and 645nm. Total chlorophyll content was calculated by employing the formula (8.02 X A_663_) + (20.2 X A_645_) and normalized to per gram of fresh weight. Water loss analysis was carried out according to Cheong *et al*. (2007) [32]. Briefly, five leaves from 3 week old WT and transgenic plants were detached and kept on laboratory bench with standard light conditions. Leaves were weighed at different time intervals to estimate the loss in fresh weight. All the experiments were repeated thrice.

### Stomata aperture assay

10 rosette leaves were detached from 4week old WT and OsPP108 overexpression plants (grown under 8 h light/16 h dark at 22°C; 70% relative humidity), and blended using a mixer/blender for 30 seconds to produce epidermal peels, in stomata opening solution (SOS: 50 mM KCl, 0.2 mM CaCl_2_, and 10 mM MES-KOH, pH 6.15), under white light (150 μmol m^-2^ s^-1^). The epidermal blends were filtered through a nylon mesh and were further incubated in SOS for 2 h under white light. For ABA treatment, the epidermal cells were incubated in SOS buffer containing 100 μM ABA, for 2h. Epidermal cells were analysed under fluorescence microscope (Olympus, BX53) in bright field and stomata apertures were examined at random fields. 30 stomata apertures were measured for WT and transgenic lines in each experiment. All experiments were repeated three times.

### Statistical analysis

All the expression, phenotypic and quantitative experiments have been replicated thrice and data have been presented as mean ± S.D (standard deviation). Two tailed student’s t-test was performed to determine the statistical significance among the samples. A p-values <0.05 was considered statistically significant.

## Results

### *OsPP108* gene is intronless and belongs to ABA related PP2Cs

**Fig 1:**
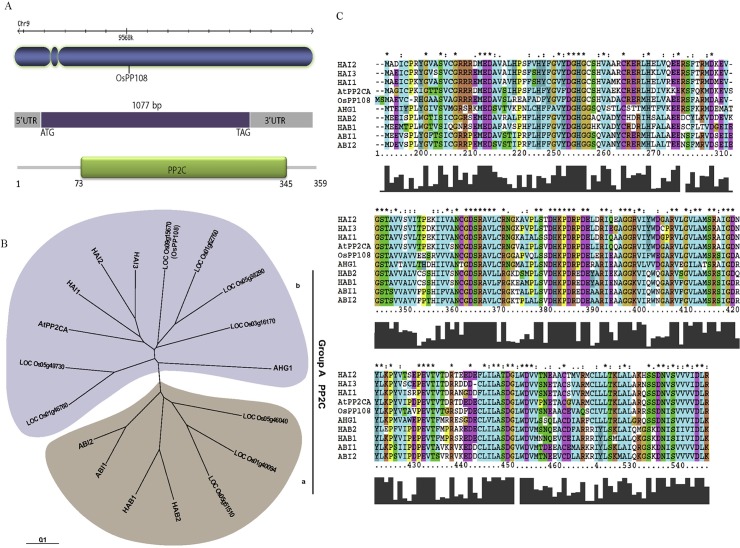
Gene, protein structure and sequence analysis of OsPP108. (A) Localization of OsPP108 gene on chromosome 9 of rice, gene structure showing that *OsPP108* is intronless and has 1077 bp open reading frame. Protein structure showing that OsPP108 protein is comprised of 359 amino acid residues and PP2C catalytic domain starts at amino acid 73 and ends at 345. (B) Phylogenetic analysis of group A PP2Cs from rice and *Arabidopsis*. Group A PP2Cs are further divided into two sub-clades a and b and OsPP108 belongs to sub-clade b. Tree was constructed with MEGA5, scale bar represents amino acid substitution per site. (C) Multiple sequence alignment of OsPP108 with the entire group A PP2Cs from *Arabidopsis*. Amino acid position is indicated at the bottom of the alignment. Multiple alignments were done in clustalX2.0.8 and manually edited to show the conservation clearly.

Gene structure analysis showed that *OsPP108* (RGAP Locus ID; LOC_Os09g15670) is an intronless gene located on chromosome 9 encoding 359 aa (amino acids) protein (Fig 1A). Domain analysis of OsPP108 protein, predicted the presence of PP2C catalytic domain, which spans 73–345 aa and authenticated the integrity of the protein. Phylogenetic analysis using domain sequences placed this gene in the group A of rice PP2Cs [6]. We performed the phylogenetic analysis only with group A PP2Cs, both from rice and *Arabidopsis* to get a more closer view, and it was found that OsPP108 falls in subgroup b of group A rice PP2Cs (Fig 1B). Previously, group A PP2Cs have been implicated in ABA signaling and responses in plants [3,11,13–15]. Therefore, we performed multiple sequence alignment of OsPP108 and clade A PP2Cs from *Arabidopsis* and it was observed that OsPP108 has high sequence homology with all the ABA related group A PP2Cs (Fig 1C). A high degree of sequence conservation with all the members of this group from two different plant species suggested possible involvement of OsPP108 in similar ABA related function and prompted us to perform in-depth functional characterization, to comprehend the role of this PP2C gene in ABA and abiotic stress mediated signaling pathway.

### *OsPP108* gene is highly induced by ABA and abiotic stresses in rice

**Fig 2:**
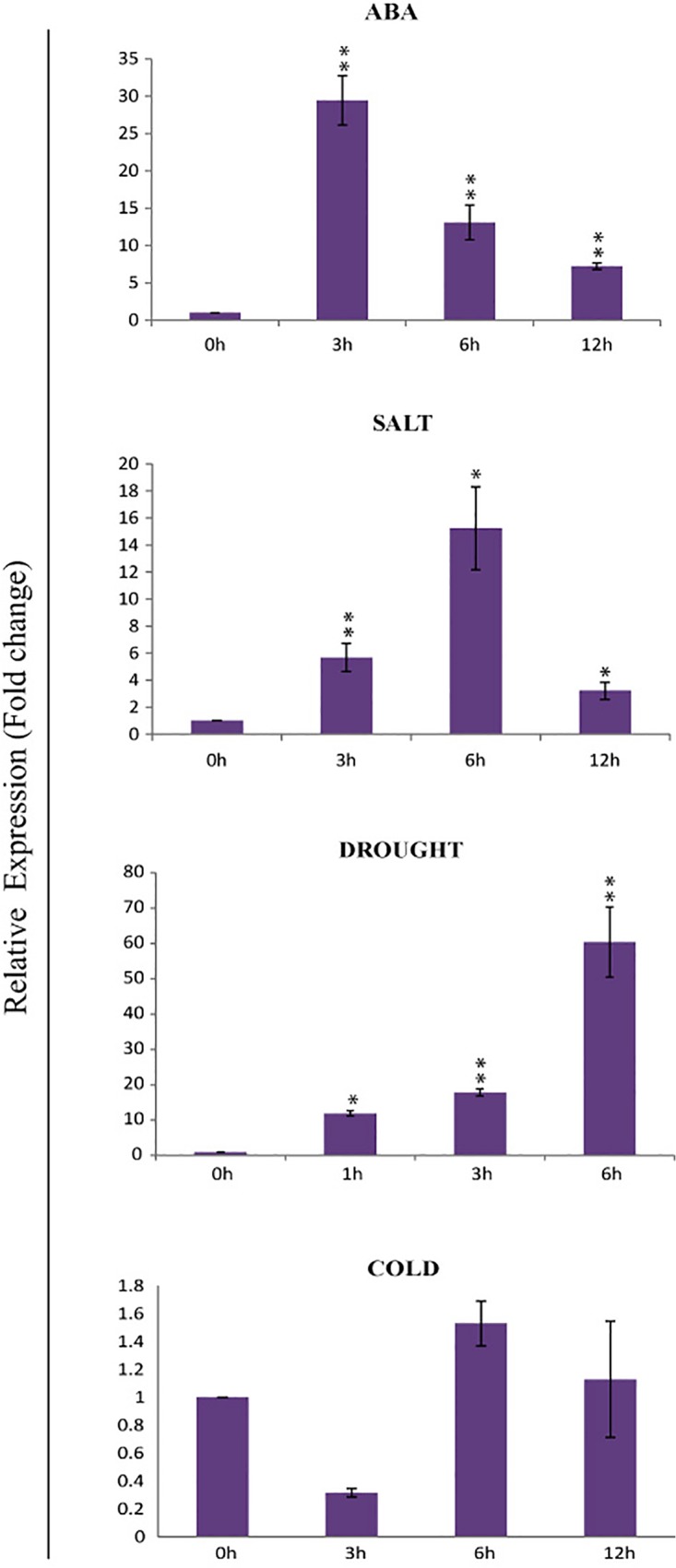
Expression profile of *OsPP108* under ABA and abiotic stresses. qPCR analysis was performed to generate the expression profile of *OsPP108* under ABA, salt, drought and cold treatment in rice seedlings. Different time period of treatment in hours is indicated on X-axis and fold change in expression level w.r.t. untreated control sample is indicated on Y-axis. Each bar represents mean value of two replicates. Standard error among the samples is indicated by error bars. * represents p-value < 0.05 and ** p-value < 0.01 for treated samples w.r.t. untreated control (0h).

In our previous study, *OsPP108* was found to be up-regulated under abiotic stresses such as salt and drought after 3 hrs of stress treatment [6]. Since *OsPP108* belongs to group A of PP2Cs, it was of interest to investigate its expression pattern under ABA and abiotic stress treatments. Therefore, we performed detail expression kinetics in rice seedlings by qPCR. Expression profile revealed that *OsPP108* was highly induced by ABA after 3 hrs of treatment and induction was about 30 fold higher in comparison to the untreated seedlings, and it subside to 12 fold and 7 folds in 6 hrs and 12 hrs, respectively (Fig 2). Similar expression pattern was suggested for *OsPP108* under ABA treatment by the microarray expression profile obtain from RiceXPro database (S1 Fig). Long term drought stress for 6 hrs leads to up-regulation of the transcript level to 60 folds with a significant up-regulation of 11 fold and 18 fold during 1 hrs and 3hrs of drought stress, respectively. *OsPP108* was also induced by salt stress treatment with approx. 6 fold up-regulation after 3 hrs, about 15 fold up-regulation after 6 hrs, and 3 fold up-regulation after 12 hours of treatment. Slight down-regulation was observed for *OsPP108* after 3 hrs cold treatment, but no significant transcript fluctuations observed after 6 and 12 hrs.

### Sub-cellular localization of OsPP108 protein

**Fig 3:**
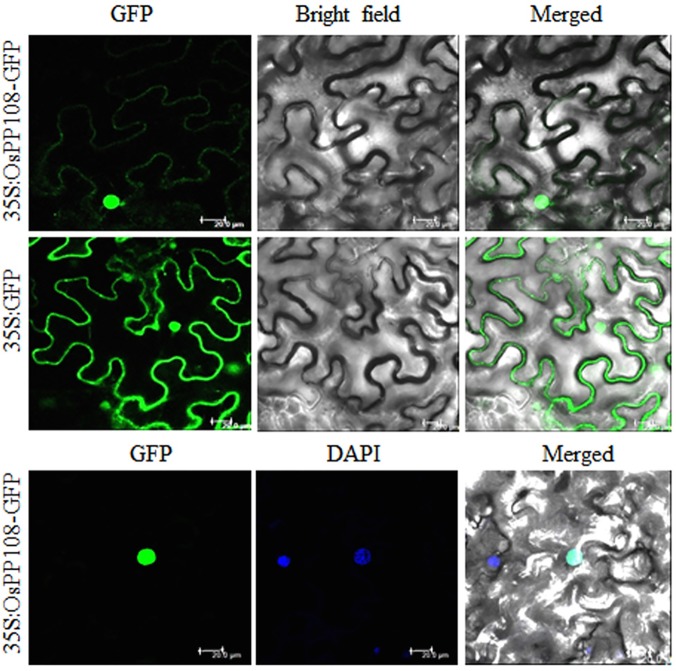
Sub-cellular localization of OsPP108 in *Nicotiana benthamiana*. Agrobacterium-infiltrated tobacco leaves expressing the GFP-OsPP108 fusion protein driven by the 2XCaMV35S promoter. Confocal images of fluorescence (green) for cell expressing OsPP108 (35S:OsPP108-GFP) are showing its distribution predominantly in the nucleus and partly in cytoplasm (upper panel). Fluorescence is distributed throughout the cells, transformed with vector only (35S: GFP) (middle panel). Co-localization of green (GFP) signal with blue DAPI organelle marker confirms the localization of OsPP108 in nucleus (lower panel). Detection of fluorescence was performed under a confocal laser-scanning microscope (wavelength: 488nm for GFP and 405nm for DAPI). Scale bar = 20 μm.

One of the key evidences for the function of a protein inside the cell is its spatial and temporal localization. To determine the sub-cellular localization of OsPP108, the coding sequence was fused with green fluorescent protein (GFP) tag and the expression was driven by 2XCaMV35S promoter. *Agrobacterium* carrying this construct was infiltrated into *Nicotiana benthamiana* epidermal peel cells to transiently express the fusion protein. Confocal microscopy analysis of *Nicotiana* epidermal cells revealed that OsPP108 protein predominantly resides in the nucleus with partial cytosolic distribution (Fig 3). Nuclear localization of OsPP108 was further supported by co-localization of DAPI with strong GFP signal in nucleus.

### Overexpression of OsPP108 leads to ABA insensitivity in *Arabidopsis*

**Fig 4:**
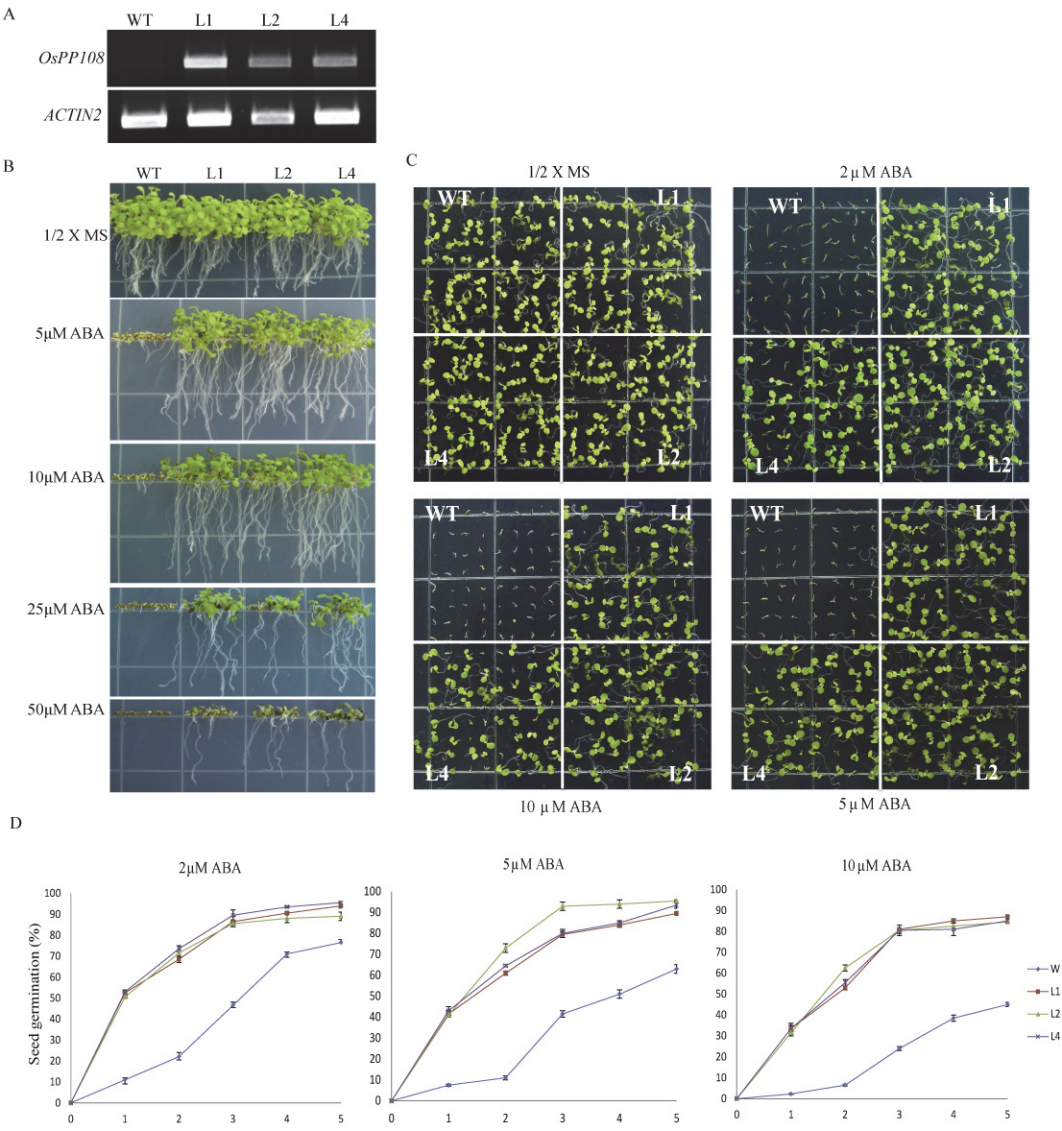
Germination and seedling growth analysis of OsPP108 overexpression transgenic *Arabidopsis* lines on ABA containing media. (A) RT-PCR analysis showing expression level of *OsPP108* in WT (Col-0) and transgenic lines (L1, L2 and L4) using gene-specific primers after 24 PCR cycles. *ACTIN2* is expressed as endogenous control in all cDNA samples. (B) Germination and vertical growth of WT (Col-0) and three OsPP108^OX^ lines L1, L2 and L4 on 1/2MS (control) and different ABA concentrations (5μM, 10μM, 25 μM and 50 μM) after 7 days. (C) Horizontal germination based growth pattern and seedling establishment of approximately 100 seeds from WT and transgenic lines on different ABA concentrations (2μM, 5μM, 10μM) after 7 days growth. (D) Seed germination percentage of WT and three transgenic lines on 2μM ABA, 5μM ABA, 10μM ABA. Germination percentage was counted for approximately 100 seeds for each genotype till 5 days of growth. Experiments were repeated three times and mean value ± SD is plotted on the graph. p-value <0.05 was observed for all the transgenic lines (L1, L2 and L4) w.r.t. WT on different ABA concentrations at different time points.

**Fig 5:**
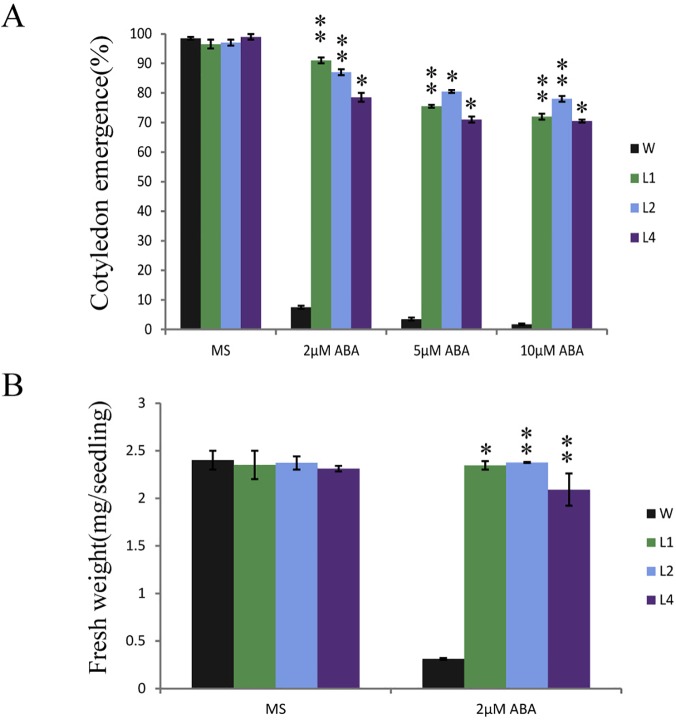
Quantitative analysis of ABA insensitive phenotype. (A) Cotyledon emergence percentage of WT and OsPP108^OX^ lines L1, L2, L4 on MS (control) media and MS media supplemented with different concentrations of ABA (2μM, 5μM, 10μM). Approximately 100 seeds were counted for each genotype and average of three observations is plotted on the graph ± SD. * p-value < 0.05, ** p-value < 0.01 shows statistically significant cotyledon emergence for transgenic lines w.r.t WT on different ABA concentrations (B) Fresh weight of 7 days old seedling grown on MS and different ABA concentrations. 15 seedlings of each genotype were recorded and average of three observations is plotted on the graph ± SD. * p-value < 0.05, ** p-value <0.01 statistically significant fresh weight of transgenic lines w.r.t WT.

The expression pattern under ABA and abiotic stresses suggested significant role of OsPP108 in ABA and abiotic stress triggered signaling in plants and prompted us for its *in-planta* functional characterization. We performed seed germination and root growth assays for three CaMV35S::OsPP108 (overexpression) lines (Fig 4A) on 1/2 MS media supplemented with different concentration of ABA (Fig 4B). Growth was monitored until 7 days and OsPP108 overexpressing plants showed insensitivity to ABA in seed germination and overall growth in comparison to WT. Insensitivity was observed until a very high ABA level (50μM). The insensitive phenotype was evaluated in term of seeds germination over a period of 5 days and all three transgenic lines could tolerate and grow much better on higher ABA level (upto 10 μM). Only 25–30% of WT seeds could germinate on this ABA concentration and almost none develop into a healthy seedling, whereas > 80% seeds of all the three overexpressing lines were germinated and developed into healthy seedlings (Fig 4C and 4D). Tolerance of OsPP108 overexpressing seeds to ABA was also estimated as full emergence of cotyledon at fourth day of growth, and all three overexpression lines had approximately 85–90% fully emerged cotyledons at 2μM ABA and 75–80% at 10 μM ABA level whereas, ~ 8% WT seeds could develop cotyledons even at 2μM ABA (Fig 5A). Fresh weight estimation after 7 days of growth at 2μM ABA showed that each seedling of three transgenic lines was ~2.4 mg, whereas WT seedling weighed only ~0.3 mg (Fig 5B), and this observation also indicated the strong insensitivity of OsPP108 overexpressing lines on higher ABA concentration. To confirm that this response is due to overexpression of OsPP108 in *Arabidopsis*, and to rule out the possibility of down-regulation of its *Arabidopsis* homologs (i.e. HAI2, HAI3, AHG1 and PP2CA) due to heterologous *OsPP108* overexpression, expression analysis was performed for all these genes in the OsPP108^OX^ lines and WT, but no significant change in expression was observed for any homolog in OsPP108^OX^ lines (S2 Fig). qPCR primers are listed in S1 Table.

### Overexpression of OsPP108 confers abiotic stress tolerance in *Arabidopsis* seedlings

**Fig 6:**
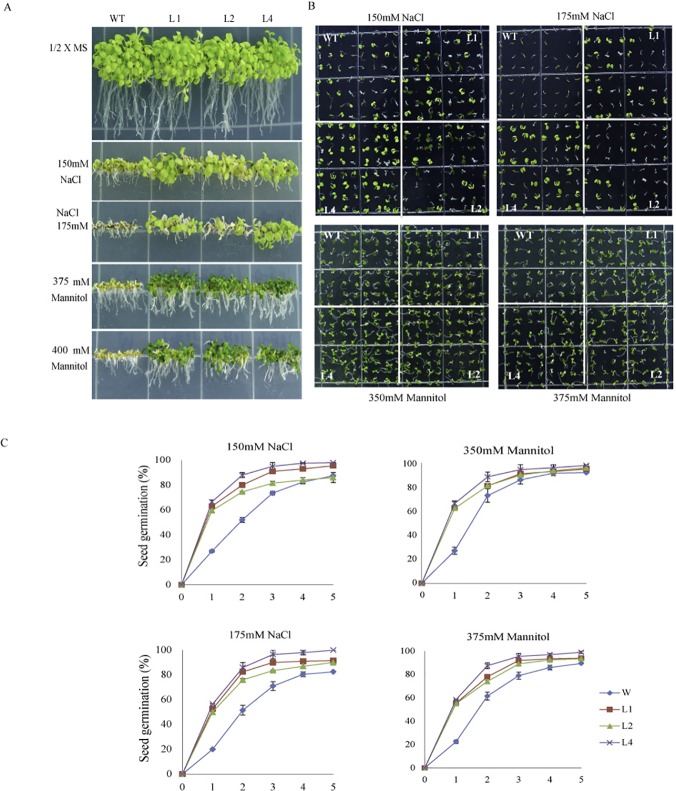
Germination and seedling growth analysis of OsPP108 overexpression *Arabidopsis* lines on salt and mannitol containing media. (A) Germination and vertical growth of WT (Col-0) and three OsPP108^OX^ transgenic lines L1, L2 and L4 on 1/2MS (control) and different concentrations of NaCl (150 and 175mM) and mannitol (375 and 400mM) after 7 days. (B) Horizontal germination and growth pattern and seedling establishment of approximately 100 seeds from WT and transgenic lines on different MS media supplemented with different concentrations of NaCl (150 and 175mM) and mannitol (350 and 375mM) after 7 days growth. (C) Seed germination percentage of WT and three transgenic lines on 150mM, 175mM NaCl and 350mM, 375mM mannitol supplemented MS media. Germination percentage was counted for approximately 100 seeds for each genotype till 5 days of growth. Experiments were repeated three times and mean value ± SD is plotted on the graph. p-value < 0.05 was observed for all the transgenic lines (L1, L2 and L4) w.r.t. WT on different stress media, at different time points.

**Fig 7:**
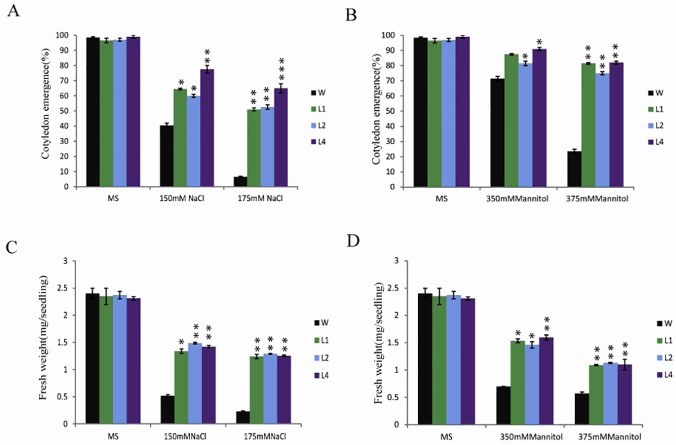
Quantitative analysis of abiotic stress tolerance phenotype. (A) Cotyledon emergence percentage of WT and OsPP108^OX^ lines L1, L2, L4 on MS (control) media and MS media supplemented with different concentrations of NaCl (150mM and 175mM) and (B) mannitol (350mM and 375mM). Approximately 100 seeds were counted for each genotype. * p-value < 0.05, ** p-value <0.01 and *** p-value < 0.005 shows statistically significant cotyledon emergence for transgenic lines w.r.t WT on different stress media. (C) Fresh weight of 7 days old seedling grown on MS and different NaCl and (D) mannitol concentrations. 15 seedlings of each genotype were recorded and average of three observations is plotted on the graph ± SD. * p-value < 0.05, ** p-value < 0.01 shows statistically significant fresh weight of transgenic lines w.r.t WT on different stress media.

Induction of *OsPP108* transcript level under abiotic stresses such as drought and salt, and insensitivity of OsPP108^OX^ transgenic plants towards ABA during seed germination enticed us to investigate the transgenic plant behaviour under abiotic stresses. Therefore, we performed seed germination and growth assays on MS media supplemented with NaCl and mannitol. OsPP108^OX^ lines could germinate and survive on high salt stress (upto 175mM) and osmotic stress (upto 400mM mannitol), and hence showed tolerance to these abiotic stresses, when compared to untransformed WT (Fig 6A). Seed germination rate was much better for OsPP108^OX^ lines than WT after 2 days of growth and more than 70% transgenic seeds could germinate on 175mM NaCl and 375mM mannitol while, ~ 45% and ~60% germination observed for WT on these stresses, respectively (Fig 6B and 6C). Analysis for cotyledon emergence after 4 days revealed that >50% transgenic seeds and only ~6% WT have emerged cotyledons on 175mM NaCl. Similarly, ~80% transgenic but only ~20% WT seeds had fully emerged cotyledon at 375mM mannitol (Fig 7A and 7B). Stress tolerance of transgenic plants was also assessed in term of fresh weight of full grown seedlings. This analysis showed that all the transgenic plants have much more weight at high salt and drought stress conditions than the WT seedlings (Fig 7C and 7D). Taken together, all these results indicated that OsPP108 overexpression confers tolerance to abiotic stresses such as salt and drought/osmotic stress in transgenic *Arabidopsis*.

### Overexpression of OsPP108 alters the expression of stress marker genes

**Fig 8:**
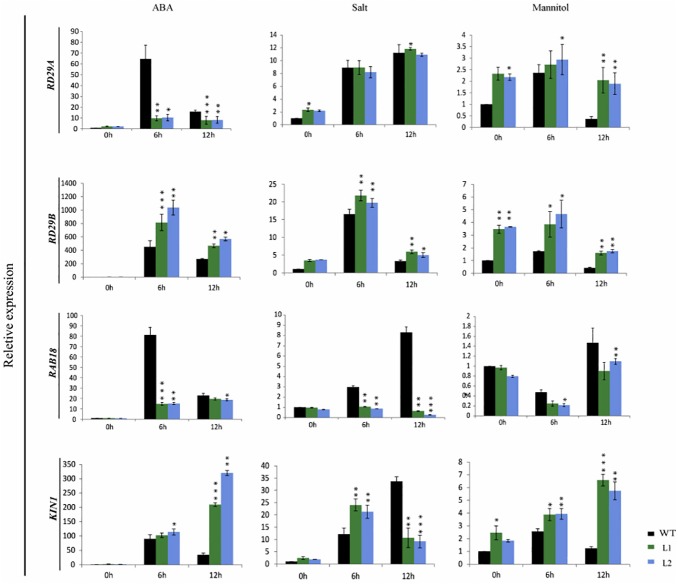
Expression profile of stress marker genes. qPCR analysis was done to generate expression profile of different stress marker genes such as *RD29A*, *RD29B*, *RAB18* and *KIN1* in WT (Col-0) and two OsPP108 overexpression lines L1 and L2 after ABA (50μM), salt (300mM) and mannitol (400mM) stress treatment. Various time points of stress treatment are indicated at X-axis and relative expression value (fold change) is indicated on Y-axis. Data from mean of two replicates is presented as columns and standard deviation among the samples is denoted by error bar. *p-value < 0.05 and **p-value- < 0.01 indicate statistically significant expression change of transgenic lines w.r.t. respective WT in different treatment and different time points.

One of the important parameters to assess the response to stress is by monitoring the expression pattern of crucial stress marker genes. Since OsPP108^OX^ plants exhibited enhanced ABA and abiotic stress tolerance, it is quite imperative to analyse the expression level of various stress marker genes in WT and transgenic plants under normal and stress conditions. Therefore, we performed qPCR based expression analysis for selected and well established stress marker genes, including *RD29A*, *RD29B*, *RAB18* and *KIN1*, using RNA from 3 week old *Arabidopsis* plants grown on MS media, and treated with different stresses (NaCl, mannitol and ABA) for 0hrs, 6hrs and 12hrs time periods. We observed that transcript level of most of these stress marker genes was higher in OsPP108^OX^ plants than WT even without any stress (Fig 8). Interestingly, *RD29A* was found to be induced at several fold higher level in WT than in OsPP108^OX^ transgenic plants, under exogenous ABA treatment. It expressed at almost similar level in WT and transgenic plants under salt stress, however expression level was slightly higher in transgenic plants under mannitol treatment. Expression level of *RD29B* was significantly higher in OsPP108^OX^ lines under ABA, salt and mannitol stresses. Notably, expression level of *RAB18* was found to decline more in transgenic plants than in WT plants under ABA, salt and mannitol stresses. Expression level of *KIN1* was consistently higher in transgenic lines under ABA and mannitol treatments, and during salt stress induction was higher in OsPP108^OX^ lines than WT after 6 hrs but higher expression observed in WT than OsPP108^OX^ lines after 12hrs of treatment. Overall, overexpression of OsPP108 in *Arabidopsis* plants enhanced the expression level of stress responsive genes without ABA and stress treatment. But under ABA and stress conditions, variable expression pattern was observed, especially for ABA related genes (*RD29A* and *RAB18*), which might be contributing to stress adaptation in overexpressing transgenic plants in ABA dependent or independent manner.

### Overexpression of OsPP108 confers abiotic stress tolerance in adult *Arabidopsis* plants

**Fig 9:**
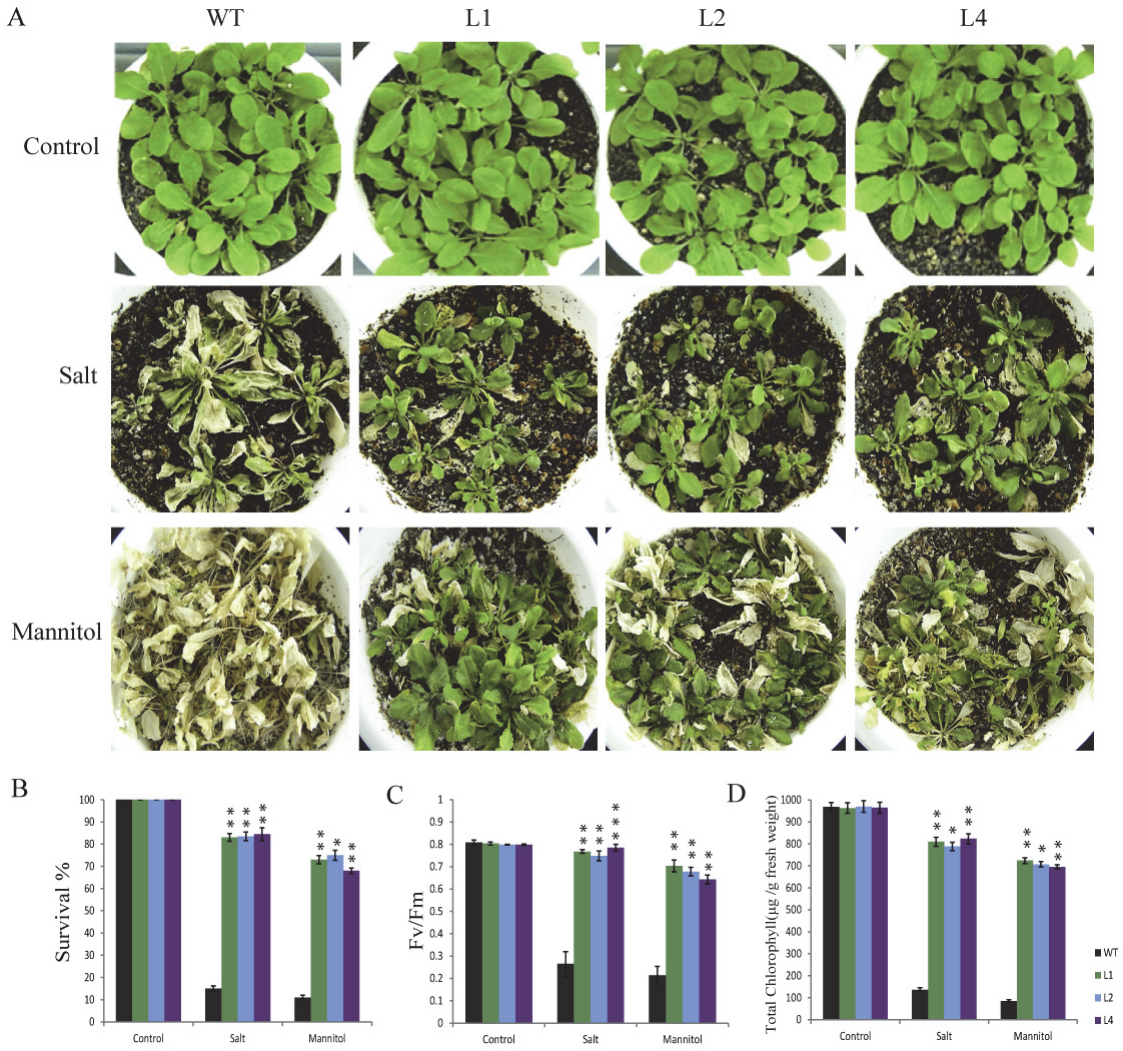
Salt and mannitol stress tolerance of adult OsPP108OX plants. (A) Phenotype of 3 week old WT and OsPP108^OX^ lines L1, L2 and L4 without stress treatment (upper row), after salt treatment (middle row) and after mannitol treatment (lower row). (B) Survival percentage of WT and different OsPP108^OX^ lines without stress and after salt and mannitol stress for 10 days. Values are mean ± SD (n = 24 plants). *p-value <0.05 and **p-value < 0.01 for transgenic lines w.r.t. respective WT in different stresses (C) Fv/Fm ratios after stress treatment. **p-value < 0.01 and ***p-value < 0.005 for transgenic lines w.r.t. respective WT, in different stresses, and (D) Total chlorophyll content after stress treatment. Data presented is mean ± SD (n = 3). *p-value < 0.05 and **p-value < 0.01 for transgenic lines w.r.t. respective WT, in different stresses.

**Fig 10:**
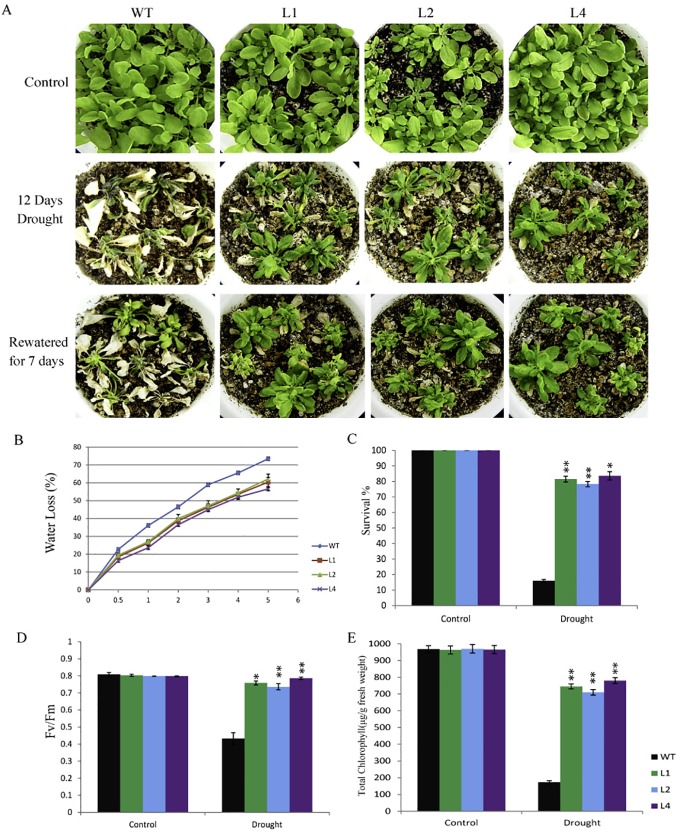
Drought tolerance of adult OsPP108OX plants. (A) Phenotype of 3 week old WT and OsPP108^OX^ lines L1, L2 and L4 without stress treatment (upper row), after drought treatment for 12 days (middle row) and after re-watering for 7 days (lower row). (B) Percentage water loss (Y-axis) from detached rosette leaves in terms of reduction of fresh weight at different time point of observation indicated at X-axis. Value is mean ± SD (n = 3). p-value <0.05 for all the transgenic lines w.r.t. WT at different time points (C) Survival percentage of WT and different OsPP108^OX^ lines without stress and after drought stress for 12 days. Values are mean ± SD (n = 24 plant). *p-value—< 0.05 and **p-value < 0.01 for transgenic lines w.r.t. respective WT (D) Fv/Fm ratios after stress treatment. *p-value < 0.05 and **p-value <0.01 for transgenic lines w.r.t. respective WT, and (E) Total chlorophyll content after stress treatment. Data presented is mean ± SD (n = 3). *p-value < 0.05 and **p-value < 0.01 for transgenic lines w.r.t. respective WT.

Adult transgenic *Arabidopsis* plants challenged with salt, drought and osmotic stresses, showed higher level of stress tolerance when compared with WT plants. 10 days after salt stress treatment most of the WT plants died and showed strong bleaching of leaves whereas, OsPP108^OX^ transgenic plants were much healthier with subtle stress susceptible symptoms (Fig 9A). Prolonged salt stress treatment (15 days) resulted in complete bleaching of leaves in WT plants while transgenic plants were growing relatively better. For salt stress, striking differences between WT and transgenic plants could be observed even after 5 days of treatment, as approximately 50% WT stems started drooping down with death of stems, leaves were wilted and siliques became yellowish, while only about 10% transgenic plant stems showed death and stress symptoms. About 80% transgenic plants could survive after 10 days of salt treatment whereas less than 15% WT plants could survive (Fig 9B). Osmotic stress was subjected by watering the plants with high concentration of mannitol solution, and observation after 10 days showed similar results as in salt stress (Fig 9A). Majority of transgenic plants survived osmotic stress while most of the WT plants died. Other quantitative parameters for assessing the stress such as total chlorophyll content and Fv/Fm ratio were also found quite high for all three transgenic lines in comparison to WT plants (Fig 9B, 9C and 9D). Similarly, OsPP108^OX^ plants could efficiently withstand the drought stress as distinctive visible phenotype was observed after 12 days of drought treatment (Fig 10A). Majority of WT plants died as stems drooped down, dried, siliques and flowers were wilted while large proportion of OsPP108^OX^ transgenic plants could withstand stress and grew healthy. Water loss measurement suggested that WT plants lost relatively more amount of water at all the time points than three OsPP108^OX^ lines (Fig 10B). Quantitative analysis revealed that more than 80% transgenic plants could survive with true leaves and vigour and they also had relatively high chlorophyll content and Fv/Fm ratio (Fig 10C, 10D and 10E). Moreover, to check the relative vigour and growth potential of the WT and transgenic plants, drought subjected plants were resupplied with water for 1 week and it was observed that plants in all three transgenic lines could recuperate from stress condition and bear healthy stems, leaf, fruits and flowers while WT plants could not regain the growth (Fig 10A and S3 Fig).

### Overexpression of OsPP108 results in ABA independent stomata movement in adult *Arabidopsis* plants

**Fig 11:**
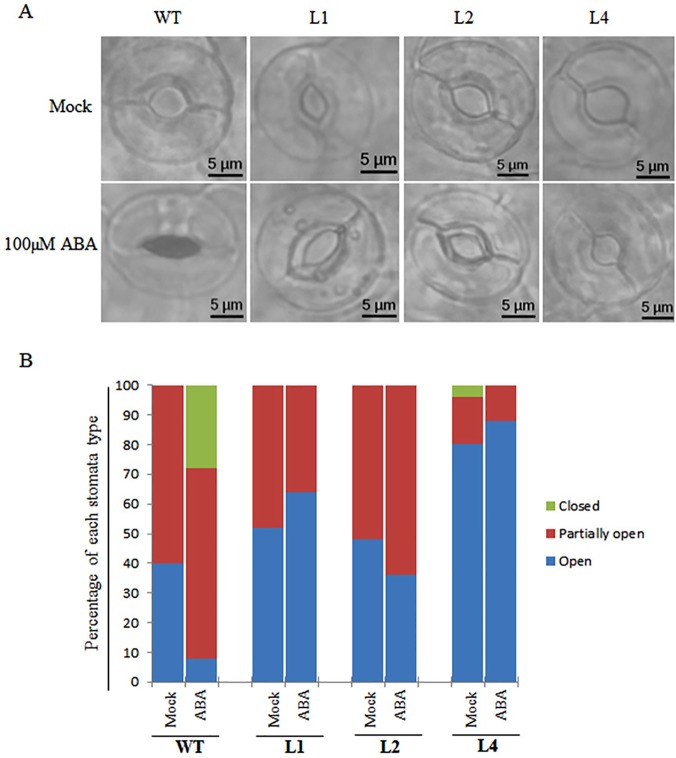
Stomata movement of OsPP108 overexpression transgenic lines under ABA treatment. (A) Stomata aperture of 4 week old WT and OsPP108^OX^ lines, L1, L2 and L4, in mock treatment (Stomata Opening Solution; SOS) (upper panel) and 100μM ABA (lower panel). 30 stomata were analyzed for each genotype and representation of those are shown in pictures (n = 3). Pictures were taken at 60x magnification in Olympus BX53 fluorescence microscope in bright field. Scale bar = 5μM. (B) Graph depicting percentage of different type of stomata (closed, partially open and open) in WT and OsPP108^OX^ transgenic in mock and ABA treatments. Stomata were grouped based on apertures sizes, i.e., open- 2.5–5.0μM, partially open- 1.0–2.5μM and closed- < 1 μM. Percentage of stomata is calculated based on the average of three independent replicate experiments.

In order to find out the causative mechanism for stress tolerance in adult plants, and its ABA dependence or independence, stomata movement assays were performed, as ABA dependent stomata closure is known to mediate drought stress tolerance. Stomata movement analysis in adult *Arabidopsis* plants revealed that in control condition (mock treatment with SOS buffer) all three OsPP108^OX^ transgenic lines have stomata aperture size similar to WT. However, treatment with 100μM ABA for 2h resulted in significant difference in stomata aperture size, and most WT stomata were closed, whereas stomata in transgenic lines exhibit ABA insensitivity and most of them remained unaffected (opened) and had aperture size similar to control condition (Fig 11A). Moreover, based on the aperture size, stomata could be divided into three groups: (i) open—2.5–5.0μM, (ii) partially open- 1.0–2.5μM and (iii) closed- < 1 μM. In WT, compare to 40% in control condition, only 8% stomata were opened after ABA treatment, approximately 60% were partially opened both in control and ABA treatment, while 28% stomata were closed after ABA treatment. In contrast, all the three transgenic lines stomata had similar percentage of open and partially opened stomata, in control and ABA conditions, and almost none of the stomata were found to be closed even after ABA treatment (Fig 11B). Therefore, these results suggest that OsPP108 mediate the stomata movement, independent of ABA pathway and OsPP108 overexpression leads to ABA insensitivity at adult plant stage also.

## Discussion

Various abiotic stresses impose adverse growth conditions for the plants in their natural environment and affect the overall productivity of crops. Stress hormone ABA is known to regulate different events of plant development in response to adverse environmental conditions and enable plants to withstand these adversities. In plants, abiotic stress triggered signaling is known to be perpetuated in ABA dependent and independent manner as expression of stress related genes can be modulated by the presence or absence of ABA [7–8, 33–34]. In *Arabidopsis*, ABA signaling triggered particularly under abiotic stresses, involve various members of group A PP2Cs as key regulators. Similar signaling cascade is also emerging in other plants species, especially model crop plant rice. Arguably, here we are presenting very first report of *in-planta* functional characterization of a rice group A PP2C, which possibly functioning in similar abiotic stress and ABA signaling module. Phylogenetic and sequence analysis revealed that OsPP108 belongs to group A of rice PP2C and its sequence is highly homologous to all the members from well-established ABA related *Arabidopsis* group A PP2C (Fig 1B and 1C). Moreover, subcellular localization in *Nicotiana benthamiana* suggested that OsPP108 protein predominantly resides in the nucleus with slight presence in cytosol as well. Similar localization have been predicted for group A PP2Cs previously, however, it was emphasized that the complex of ABA signaling components (PYR/PYL receptors, PP2Cs and Ser/Thr Kinase) is formed in the nucleus to regulate ABA mediated gene expression [21, 24]. With these clues in mind, we performed detailed expression analysis for *OsPP108* under ABA and abiotic stresses such as salt, drought and cold. To our anticipation, this gene was highly induced under exogenous ABA treatment, salt and drought stresses at different time periods, while expression was insignificant under cold stress. Moreover, *in-silico* promoter analysis revealed the presence of twelve ABA responsive elements (ABRE) and one motif IIb (CCGCCGCFCT) which is also an ABA responsive element, in 1kb upstream sequence of *OsPP108* (S4 Fig). These important observations indicated towards the vital functional role of this rice PP2C in ABA and abiotic stress signaling network. To validate this assumption, three independent homozygous *Arabidopsis* transgenic lines constitutively expressing *OsPP108* were analysed for ABA sensitivity and tolerance on MS based media. Transgenic lines showed marked difference when compared to WT, and all three transgenic lines could not only germinate but grew and developed into healthy seedlings at higher ABA concentration as much as 50μM, while WT could not even germinate after 2μM ABA (Fig 4B). This drastic phenotypic difference was also supported by various quantitative parameters such as seed germination rate, seedling fresh weight and cotyledon emergence. The genetic evidence suggested that OsPP108^OX^ plants are highly insensitive to ABA, and like other group A PP2Cs, this rice PP2C also act as negative regulator of ABA signaling. This ABA insensitive phenotype of OsPP108^OX^ plants is quite comparable with *snrk2*.*2/2*.*3/2*.*6* triple mutant plants on high level of ABA [35–37]. SnRK2 kinase members 2.2, 2.3 and 2.6 work in ABA signaling as positive regulators. Previously, various *Arabidopsis* group A PP2C members have been characterized in ABA signaling. HAB1 overexpressing transgenic plants also showed high ABA insensitivity in seed germination and root growth at higher exogenous ABA levels while *hab1-1* mutants showed high ABA sensitivity [13, 38]. Similarly, *Arabidopsis* plants overexpressing AtPP2CA exhibited high ABA insensitivity in seed germination and stomata aperture closure, whereas its knockout mutants showed opposite phenotype [39], and similar results were also observed by Yoshida and co-workers with overexpression and knock-out mutant plants of AtPP2CA [3]. Also, different double and triple mutants of ABA related group A PP2Cs such as, ABI1, ABI2, HAB1 and PP2CA exhibited highly ABA sensitive phenotype in seed germination, root growth and overall development [40]. Recently, another member of *Arabidopsis* group A PP2Cs called HONSU was found to negatively regulate ABA signaling to regulate seed dormancy, and its overexpression resulted in phenotype similar to OsPP108^OX^ at higher concentration of ABA, while mutant exhibited ABA sensitivity [41]. Altogether, our study suggests that OsPP108 can be placed in the ABA signaling module similar to other group A PP2Cs in *Arabidopsis* and rice. Germination based assays on high salt and osmotic/drought media showed that OsPP108^OX^ transgenic lines could tolerate abiotic stresses much better than WT plants (Fig 6A and 6B). Stress tolerance of transgenic lines was evident from better overall shoot and root growth, better germination rate, cotyledon emergence rate and fresh weight of the seedlings on NaCl and mannitol supplemented MS media (Fig 6C and Fig 7). Saez *et al*. (2004)[13] also showed that HAB1 overexpressing lines showed better germination and seedling growth rate on higher ABA, salt and mannitol media while its knock-out mutants showed reverse pattern. Hyperosmotic and salt stress are known to increase the production of ABA in plants [34, 42–43] and plants, which show hypersensitivity to ABA may also exhibit hypersensitivity to salt and osmotic stresses either due to increased sensitivity to ABA or increased production of ABA [33–34, 44–45]. In other words, abiotic stress responses may be controlled by ABA dependent or ABA independent pathway. Based on our result, we speculate that the abiotic stress tolerance of OsPP108^OX^ lines might be through ABA independent pathway, however, this needs further experimental verification. To support this assumption, a recent report in *Arabidopsis* has shown that the group A PP2Cs especially, three highly ABA induced (HAI) PP2Cs, which are also the closest homologs of OsPP108 in *Arabidopsis*, were highly induced under low water potential stress whereas, expression of many ABA receptor i.e. PYLs was down-regulated [15]. Moreover, it has been reported that overexpression of PYL5 and PYL9 resulted in ABA hypersensitivity [17, 38] and it was hypothesized that the regulation of PYL expression can be a converging point where crosstalk between ABA dependent and independent signaling factors might take place [15]. Moreover, in our analysis, expression level of stress marker genes such as *RD29A* and *RAB18* was found to be declined significantly in OsPP108^OX^ lines in comparison to WT, under exogenous ABA treatment. These stress marker genes are known to be regulated through ABA signaling, during abiotic stress responses [46–49]. Notably, *RD29A* expression was higher in transgenic plants than WT under mannitol treatment, while *RAB18* was down-regulated not only under ABA, but also in salt and mannitol stresses (Fig 8). This behaviour could be due to the presence of specific ABA responsive element in promoter of *RAB18* [8, 50–51] and presence of both drought responsive elements and ABA responsive elements in promoter of *RD29A* [48]. Previously, expression was found to be declined for *RAB18* and *RD29A* stress genes where PP2C transgenic plants exhibited ABA insensitive response and vice-versa [13, 52–53]. However, variable expression pattern for stress markers such as *RD29A*, *RD29B* could be attributed to multi-layers of gene expression regulation. Key transcription factors such as DREB and AREB, which ultimately control the expression of stress genes such as *RD29A* and *RD29B*, might be variably (and indirectly) controlled by OsPP108 or other components such as SnRK2s (since SnRK2s are downstream to PP2C), which are known to regulate the stress responsive transcription factors. Moreover, at many instances gene expression pattern have not been found to be directly correlated with the observed phenotypes [54–55], and post translation modification might also lead to the resulting effects. It can be speculated that OsPP108 might interact and regulate some critical components of ABA signal transduction pathway such as ser/thr kinases, especially SnRK2 [33, 56–57] and CIPKs [33, 45, 55], which have been implicated in ABA, salt and osmotic stresses and could influence the expression of stress responsive genes to regulate the physiological processes. Previously, PP2Cs have been found to physically interact and form complexes with kinase such as CIPK15/PKS3 [58–59] and OST1/SnRK2.6 [2, 3, 5] in negative feedback loop to regulate ABA signaling. Guo et al. (2002) [58] demonstrated that RNAi knockdown lines of CIPK15/PKS3 were sensitive to ABA in germination and double mutants of *pks3* with dominant *abi1* and *abi2* mutants could recapitulate the phenotype towards WT and similar to *abi1* and *abi2* mutant phenotype. At adult stage most of the OsPP108^OX^ transgenic plants could withstand the stress conditions and found to retain high level of chlorophyll. Chlorophyll fluorescence is a vital parameter to gauge early signs of stress and hence considered as a suitable way to assess the stress tolerance in plants [60]. Therefore, chlorophyll fluorescence was measured to estimate the photochemical efficiency or photosynthetic potential of transgenic and WT plants in terms of Fv/Fm ratios. Under normal conditions, there was no significant difference but under salt, drought and osmotic stresses transgenic plants found to have far better photosynthetic potential (Fig 9C and Fig 10D). Better tolerance of transgenic plants to drought stress was further supported by water loss assay where transgenic plants were found to retained higher amount of water at different time points than WT plants. Reduced water loss can also be understood as enhanced water retention ability or osmotic potential, which help the cell to maintain regular turgor and prevent membrane damage. ABA induced stomata closure is an important adaptive response when plant encounters drought stress, and results in reduced water loss [24]. Therefore, we analyse the stomata aperture size at the adult stage of OsPP108^OX^ plants. Consistent with the ABA insensitivity at the seed germination level, adult plants also showed ABA insensitivity and most stomata were unaffected and remained opened or partially opened, after high concentration ABA treatment (Fig 11). This observation suggested that OsPP108 might regulate abiotic stress (especially drought) tolerance through ABA independent mechanism. Notably, an inward rectifying potassium channel, KAT1 is known to be involved in stomata movement in *Arabidopsis* and SnRK2E/OST1 can target KAT1 and can affect its channel activity through phosphorylation at Thr306 [61]. Therefore, ABA activated SnRK2 may promote stomata closure by negatively regulating KAT1 activity [62]. However, in case of ABA insensitive PP2C such as OsPP108, SnRK2 may remain inactive as its inhibition by PP2C could not be released due to absence of PP2C interaction with ABA activated ABARs and might result in failure of stomata closure. This observation requires clarification that how OsPP108^OX^ plants can show reduced water loss when their stomata are ABA insensitive and mostly opened. We speculate that OsPP108 transgenic plant may not be completely ABA insensitive at molecular level, as expression level of few stress marker genes was still higher in OsPP108^OX^ plants than WT under ABA treatment (Fig 8), and might be contributing to stress tolerance.

OsPP108 might be involved in osmoregulatory function, maintenance of high water potential in dehydration conditions, independent of ABA pathway, as proposed for its close homologs HAIs in *Arabidopsis* [15]. Alternatively, OsPP108 might be regulating TFs such as WRKY46, which has been found to regulate osmotic/drought stress response and stomata movement independently [63]. WRKY46 was found to control the expression of genes coding LEA (late embryogenesis abundant) proteins, which participate in cellular osmoprotection during osmotic/drought stress, and genes involve in redox homeostasis and protection against oxidative damage. Moreover, in WRKY46 overexpressing plants stomata movement was controlled by light dependent starch metabolism in guard cells, and this process was independent of ABA. Recently, similar mechanism for drought stress tolerance in rice was shown by a PP2C. Overexpression of PP2C, *OsPP18* led to drought and oxidative stress tolerance in transgenic plants through controlling gene expression of ROS scavenging enzymes and ROS homeostasis, in ABA independent manner [64]. However, these speculations require further experimental support.

Previously, not many PP2Cs have been implicated in salt stress responses in plants; a maize member ZmPP2C found to negatively regulate salt stress response in *Arabidopsis* [65]. As an initial evidence, rice leaf disc based stress tolerance assay also revealed that OsPP108 regulate salt and drought stress signaling positively in rice at the adult stage of development, and overexpression of OsPP108 confer enhance stress tolerance (data not shown).

In conclusion, OsPP108 is a rice group A PP2C and like other PP2Cs of this group from rice and *Arabidopsis*, it negatively regulates ABA signaling. OsPP108 overexpression results in high ABA insensitivity at the seed germination, early seedling development and stomata closure at adult stage. On the other hand, it positively regulates salt, drought and osmotic signaling as its overexpression lead to enhance stress tolerance in *Arabidopsis*, at seed germination and adult stage. Transgenic rice plants overexpressing OsPP108 are expected to show similar responses to ABA and abiotic stresses and may provide an answer to plants vulnerability in the natural environment to these adverse conditions, and ultimately result in higher crop yield and better productivity.

## Supporting Information

S1 Fig Microarray expression profile of OsPP108 under ABA treatment from RiceXPro database in root and shoot. Bars representing expression profile of three and two replicates in root and shoot, respectively for mock (control) and ABA treatment for different time points, indicated at X-axis.
(EPS)

S2 Fig Expression profile of OsPP108 homologs AHG1, PP2CA, HAI2 and HAI3 in Arabidopsis. qPCR analysis was done using gene specific primers in WT and OsPP108^OX^ lines 1, 2 and 4.
(EPS)

S3 Fig Phenotype of WT and OsPP108^OX^ lines L1, L2 and L4 plants, after 7 days of re-watering post 12 days drought treatment. (EPS)

S4 Fig 1 kb upstream promoter sequence of OsPP108 extracted from PlantCARE database. 12 ABRE elements were marked in the promoter sequence are highlighted with yellow color.
(EPS)

S1 Table Primers used for qPCR expression analysis. (XLSX)
